# Delayed diagnosis of anorexia nervosa following childhood cancer treatment: a case report and literature review

**DOI:** 10.1186/s40337-026-01538-5

**Published:** 2026-03-02

**Authors:** Hannah Sophie Fuchs, Carolyn Nahman

**Affiliations:** University of Oxford, Oxford, UK

**Keywords:** Anorexia nervosa, Paediatric oncology, Cancer survival, Eating disorders, Diagnostic delay, Wilms tumour, ARFID (avoidant restrictive food intake disorder), Adolescent mental health.

## Abstract

**Background:**

Paediatric oncology treatment frequently affects appetite and eating behaviours, yet there is limited evidence regarding the incidence and recognition of eating disorders among childhood cancer survivors. Misinterpretation of eating-related symptoms may delay appropriate psychiatric care.

**Case presentation:**

We describe a 15-year-old male survivor of stage IV Wilms tumour, previously treated with chemotherapy and nephrectomy, who presented during oncology follow-up with weight loss, food restriction, and excessive exercise. Despite fulfilling diagnostic criteria for anorexia nervosa, his symptoms were initially attributed to post-traumatic stress and disease recurrence, resulting in a four-month delay in referral and treatment. On eventual admission, he met multiple Medical Emergencies in Eating Disorders (MEED) red criteria. Following inpatient treatment with nutritional rehabilitation and psychological support, the patient made a full recovery and remains in remission from both his malignancy and eating disorder.

**Conclusions:**

This case illustrates diagnostic overshadowing of anorexia nervosa in paediatric oncology survivors, a population potentially at increased risk for disordered eating due to treatment side effects, body image changes, and psychological trauma. Routine screening for restrictive eating, avoidant restrictive food intake disorder (ARFID), and food-related anxieties during oncology follow-up may facilitate early recognition and intervention. Increased awareness of eating disorders as possible late effects of cancer treatment is warranted among oncology and mental health professionals.

## Case report

A 15-year-old male presented for a routine follow-up paediatric oncology appointment with loss of appetite, 5 kg weight loss, tightness in his chest, and feeling not quite himself. He had previously been under care by the paediatric oncology team for a stage IV left Wilms tumour, diagnosed at age two, and treated with vincristine, actinomycin D, and doxorubicin chemotherapy as well as a left nephrectomy, achieving complete remission. Presuming a disease recurrence, investigations for bloods, chest x ray, urine dip stick and abdominal ultrasound analysis were arranged, which were normal, and a three-month follow-up with repeat investigations was arranged. At this follow-up appointment, the patient had lost a further 4 kg, and displayed clear signs and symptoms of anorexia nervosa, including excessive exercise, feeling like he does not deserve food, concerns about becoming “fat and lazy” and eating more than his peers. He also started developing panic attacks. At this clinic visit heart rate was 50 bpm and his blood pressure 93/55 mmHg. He had also developed cold intolerance.

This prompted an urgent Child and Adolescent Mental Health Service (CAMHS) referral, however, was downgraded to routine and delayed for further three months as his symptoms were hypothesised to represent a post-traumatic stress disorder rather than an eating disorder. A cardiology referral had also been made, where the patient was discharged as he was, albeit bradycardic, deemed “fine from a cardiac point of view”. This bradycardic electrocardiogram was reviewed by another CAMHS psychiatrist, who identified the acutely life-threatening nature of this patient’s eating disorder, meeting multiple red criteria of the Medical Emergencies in Eating Disorders (MEED) criteria [1].

The patient was then admitted to a paediatric ward, at which point the patient had lost 12 kg relative to his base line, weighing 59 kg at a height of 194.4 cm, had a heart rate of 36 bpm and a blood pressure of 88/51mmHg. The patient’s height for age was around the 99th percentile from age 12, surpassing the 99.5th percentile by age 15. Before his illness, the patient’s weight progressed along the 90th percentile, but had now dropped below the 50th percentile (95% weight for height [WFH] down to 81% WFH). He commenced a meal plan and completed an emergency CAMHS assessment followed by admission to an inpatient CAMHS unit with a specialist eating disorder support.

According to National Institute for Health and Care Excellence (NICE) guidelines, the patient was treated with Family Therapy for Anorexia Nervosa (FTAN). The traditional stages of FTAN were followed and he rapidly progressed to stage 3 and through stage 4. Psychoeducation and engagement formed an important part of the treatment, with the patient appreciating factual scientific explanations for urges to exercise and distress caused in early stages of improving his nutrition. He and his family were emotionally attuned and discussions around the trauma around his cancer diagnosis and treatment, the impact of parental illness and COVID-19 lockdown on him and his family. His parents supported both the refeeding and helping the patient find rest. Education was important for him and within his school he was a valued, likeable pupil. His school facilitated an early re-integration, and he also received support from trusted peers. He made a full recovery, regaining a healthy weight once again around the 90% WFH (with additional growth in height), and he is now thriving at university. He remains in remission from both his cancer and his eating disorder.

In summary, this patient developed an eating disorder in the context of a diagnosis of cancer as a child and oncology treatment, with significant delays to diagnosis despite meeting all core eating disorder diagnostic criteria. In his oncology follow-up appointments, he was described as a “robust lad” with a strong can-do attitude, he is high achieving both in academics and sports, with a mature personality. According to his family he never expressed negative emotions and felt like he needed to be positive. He also described a need to control and plan everything. His eating disorder was likely precipitated by adopting a “healthy eating strategy” to keep healthy secondary to his previous cancer diagnosis, the COVID-19 pandemic, and illnesses which occurred with both parents around similar times.

The young man and his family were incredibly resourceful and motivated, and he did well after eating disorder treatment and made a full recovery and he is now a young adult, in higher education and thriving in all areas of his life.

**Fig. 1:**
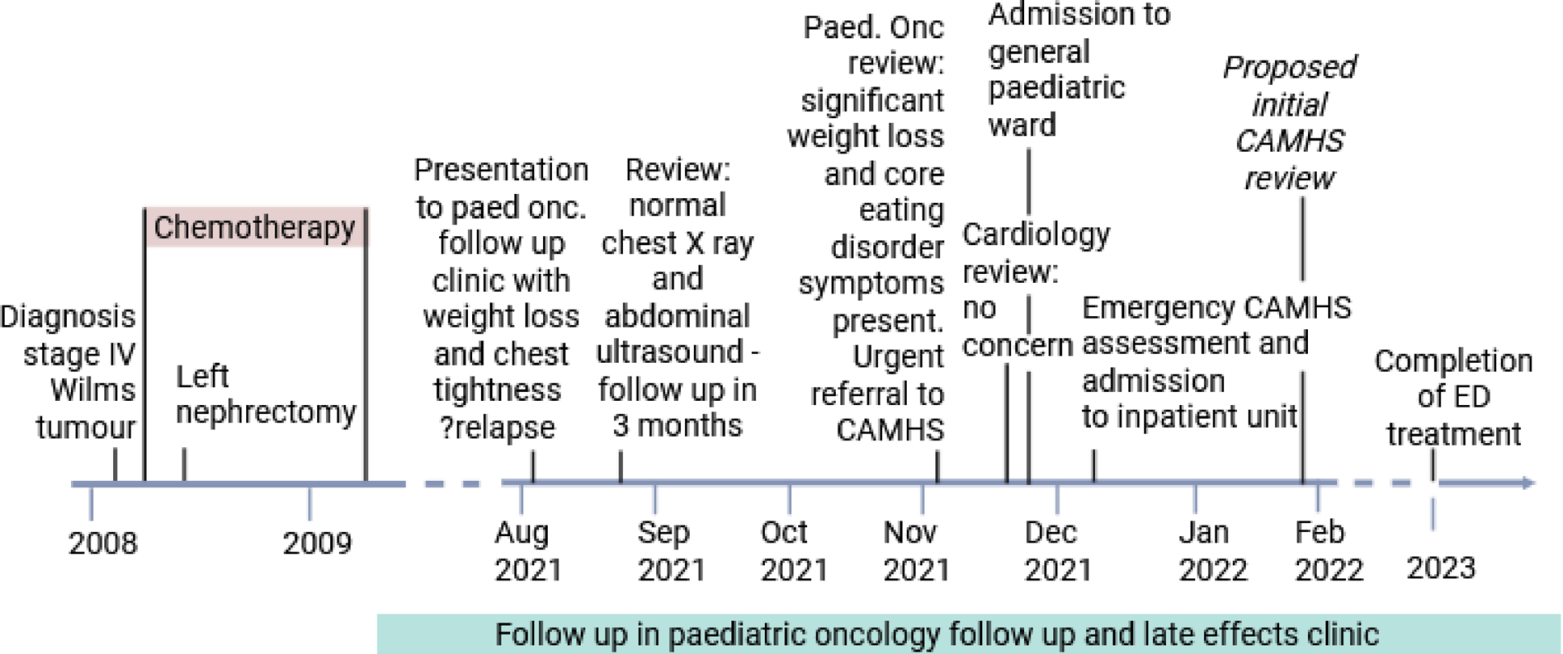
Timeline of presentation and treatment of Wilms tumour and anorexia nervosa

## Literature review

Eating disorders span the interface between endocrinology, neurobiology, and psychiatry. Paediatric neurological conditions such as craniopharyngiomas [2, 3], or Prader-Willi syndrome [4, 5] are well known to cause changes in appetite and eating patterns. Brain tumours can thus mask as eating disorders. Case reports describe hypothalamic tumours [6, 7], pontine tumours [8], craniopharyngiomas [9], and other central nervous system (CNS) tumours [10–14] being initially investigated as eating disorders, leading to significant delays to the diagnosis and treatment of underlying malignancy. Beyond weight loss, many of the described patients display classic eating disorder behaviours, such as dissatisfaction with body shape [9], food restriction or refusal [6, 7, 13, 14], requiring strict rituals and routines to eat [8] and associated psychiatric symptoms such as mood changes [6, 7, 9], illustrating similarities in presentation of eating disorders and CNS disease beyond frank weight loss with changes also to behaviour and food- and body-related cognition.

Beyond the hypothalamus and higher eating control centres, endocrine mediators of appetite and weight, of which leptin is perhaps the best studied, are dysregulated in eating disorders such as anorexia nervosa [15–18]. Various studies report similar changes in cancer patients [19–21], with proposed overlapping physiological and psychological consequences [18, 22]. Weight loss itself causes profound and long-lasting psychological distress even in people without pre-existing disordered eating, which has been known since the Minnesota Starvation Experiment [23].

The prevalence of undernutrition in children with cancer, particularly solid tumours like Wilms tumour, is high [21, 24], and related to worse health-related quality of life [24, 25]. Thus, beyond structural changes imposed by CNS mass lesions and abnormalities to endocrinological and metabolic appetite regulation, the weight loss associated with a malignancy or the oncological treatment thereof, may itself contribute to disordered eating thoughts and behaviours. Consequently, further case reports describe non-CNS tumours presenting as eating disorders [12, 26, 27].

Chemotherapy- and radiotherapy-associated side effects that interfere with food intake are well described [28–30] and occur frequently: in a cohort study of 160 children with cancer undergoing chemotherapy, nausea (44.7%) and lack of appetite (39.6%) were some of the most common, and most distressing symptoms. Children also experienced vomiting (27.7%), mouth sores (13.9%), difficulty swallowing (12.6%), and a subjective change in taste (16.5%) [31]. An objective assessment of smell and taste in children with cancer showed significant alteration following chemotherapy cycles [32]. A qualitative analysis into reasons for eating problems in children undergoing cancer treatment highlighted pain, altered taste, lack of appetite, nausea and vomiting, fever or feeling ill, aversion to hospital food, the ward environment; and inadequate food intake causes major child and parental concern and distress [28, 33].

Corticosteroids, which are widely used in oncology treatment, have well described behavioural and psychiatric adverse effects, including anxiety, depression, and psychosis [34–36], as well as body dysmorphia precipitated by increased appetite, weight gain and change of facial features [36].

Further, the significant psychological impact of experiencing oncological disease and treatment on paediatric patients and their family is well-document [37, 38] and psychiatric effects can be long-lasting. With a median follow-up period of 21 years, long-term outcomes in paediatric leukaemia and lymphoma survivors revealed worse scores of physical and social function, pain, and general physical and mental health: higher rates of anxiety, depression, fatigue, insomnia, obesity, chronic pain, and substance abuse were observed [39].

Disordered eating often represents a dysfunctional coping strategy [40, 41], and in the context of cancer treatment, eating problems such as food refusal, have been described as a means to gain control of over a distressing situation [31]. Together with the neurobiological links between malignant disease and food and appetite regulation, and the impact of oncological treatment on diet, appetite, and weight, this suggests paediatric cancer patients may be at a high risk of developing eating disorders.

The increased incidence of body image disturbance and eating disorders in non-oncological paediatric serious illness where treatment includes dietary changes, such as cystic fibrosis [42–44], coeliac disease [42, 45–47], and especially diabulimia in the context of diabetes mellitus [42, 48–50], is known. Nonetheless, the literature on the incidence of eating disorders in paediatric oncology patients is very sparse.

A PubMed search using the Mesh terms (((“Neoplasms“[Mesh]) AND “Feeding and Eating Disorders“[Mesh]))) AND (“Adolescent“[Mesh] OR “Child“[Mesh]), as well as additional searches under the title “paediatric cancer eating disorders” and “paediatric cancer survivor mental health” conducted between 10.12.24 and 29.12.24 yielded a total of 706 articles. Of those, 359 were primary articles written in English or German reporting physical or mental health-related outcomes in paediatric cancer patients. Of these, 24 studies reported eating or feeding problems. Excluding case reports 13 studies remained and only seven studies formally assessed for the presence of eating disorders or eating disorder behaviours. The vast majority of studies assessing mental health outcomes in paediatric cancer survivors do not mention eating disorders [51–68], incl. a study specifically studying the long-term psychological consequences of Wilms tumour [69].

Despite the apparent scarce literature, the studies that do exist suggest that disordered eating may be widespread among paediatric cancer survivors. A retrospective study assessing long-term health outcomes in 112 children with haematological malignancies who had undergone haematopoietic stem cell transplantation reported psychological disturbance in 49.5% of patients, and 54% of patients with long-term complications, with 16 (14.3%) showing eating disorder behaviours. 15 patients developed anorexia and one developed hyperphagia [70]. A cohort study of 15 adolescents undergoing cancer treatment, and 39 young adults who had completed treatment, reported psychosocial distress during treatment as well as after, with frequent eating problems encountered by 53% of patients on treatment, and 20% after treatment. This was highly associated with overall life satisfaction [71].

Three further studies investigated the presence of psychiatric disorders post treatment with a small proportion of survivors developing eating disorders. Semi-standardised interviews in 130 survivors of non-leukaemia childhood cancers comprehensively reported two incidences of eating disorders, which is not significantly different from the national eating disorder incidence [72]. A small cohort study of 34 medulloblastoma survivors reported one case of bulimia nervosa [73]. A large case and sibling control cohort study with 13,860 paediatric cancer survivors found increased risk for “somatisation/eating disorders” in female survivors, however there was no subgroup analysis looking at eating disorders alone [74]. One study specifically investigated the presence of feeding difficulties and eating disorders in paediatric patients with cancer using the Identification and Management of Feeding Disorders questionnaire, with 92% of children displaying one or more feeding difficulty or eating disorder, namely limited appetite, selectivity, or fear of feeding [75]. A final study aimed to implement screening for eating disorders in adolescent and young adult cancer survivors in an oncology follow-up clinic in Minneapolis, identifying 11 eating disorders among 163 eligible patients (6.7%), a rate more than double the general population incidence reported in the United States (2.7%) [76]. In summary only a total of seven studies investigated the presence of eating disorders of childhood cancers survivors with substantial variation in study type, cancer type, sample size, and definition of eating disorders, with eating disorders being the focus of investigation only in two studies [75, 76].

## Conclusions

This case report and literature review highlights a significant gap in our understanding of the link between eating disorders and paediatric malignancy, which can often present with similar symptoms. Weight and appetite changes might mask eating disorders in patients with cancer and other severe diseases. Here we describe a 15-year-old patient with weight loss and a delayed diagnosis of and access to treatment for anorexia nervosa, due to diagnostic overshadowing with symptoms being initially interpreted as disease relapse and post-traumatic distress. Review of the literature reveals that eating disorders are currently less well researched in the field of paediatric oncology compared to paediatric endocrinology and gastroenterology. This may possibly link to survival and gratitude bias, a common attitude specifically towards cancer survivors, both in personal and professional relationships. While post-traumatic growth attitudes predict higher quality of life in paediatric cancer survivors [77], this has the potential to mask adverse mental health outcomes.

Avoidant Restrictive Food Intake Disorder (ARFID) post cancer is poorly researched and consideration and further research is needed to look at potential food aversion, vomit phobia and other symptoms related to treatment side effects which might create long term fears. Additionally, the uncertainty around cancer relapse might create an unhealthy relationship with healthy living and healthy eating. Perceived stress, particularly with co-existing irrational health beliefs, is associated with problematic eating [78]. The challenges of adolescence, including identity finding, contribute to this age group being at risk for the onset of eating disorders [41, 79, 80]. Negotiating adolescence with the additional stressors of having previously experienced a severe illness alongside adolescence as well as medical demands which include medical evaluations, illness related stigma might exacerbate disordered eating as a maladaptive coping strategy [81].

Research into psychological outcomes and relationship with food and nutrition both during and after paediatric oncology treatment are currently inadequate and are necessary to understand psychological health, mental disorder, eating disorders and relationship with food and specific individual risk factors after treatment of cancer. Qualitative studies with survivors and their carers would help understand how oncology treatments impact mental health.

Within young people who complete treatment for cancer, many would benefit from psychological support around return to normal life, building a positive relationship with food, and coping with uncertainty around relapse. Paediatric oncology follow-up appointments offer critical opportunities to explore psychological wellbeing and potential detection of mental health problems and difficulties around eating/relationship with food, particularly when offered in regular intervals throughout stages of development and adolescents, as screening immediately post-treatment may be too early to identify specific areas of difficulty in coping.

Finally, CAMHS services are overloaded and not always able to consider individual needs but should remain sensitive to risk of adverse mental health outcomes after serious illness. An assumption that a disordered eating presentation was directly related to potential recurrence of, or trauma related to previous physical illness, as happened with our patient, lead to delayed presentation. This poses a risk for missed or delayed diagnosis of an eating disorder in this at-risk population. This highlights the limitations of CAMHS single point of access triage systems, and the need for improving access to treatment, as well as the lack of awareness about the link between oncology and eating disorders.

The expansion of child and adolescent consultation liaison psychiatry services could facilitate communication between paediatric medical and psychiatric care and improve patient outcome. Paediatric oncology services should be aware of the potential for changes in a relationship with food and eating and the potential for early intervention to prevent eating disorders and consider these as differentials in patients presenting with distress, behavioural change, and weight loss during follow-up. As demonstrated in this case report, oncology and late effect clinics provide important opportunities for the detection of psychiatric illness, including eating disorders [76].

## Data Availability

No datasets were generated or analysed during the current study.

## Abbreviations

ARFID: Avoidant restrictive food intake disorder
CAMHS: Child and adolescent mental health service
CNS: Central nervous system
FTAN: Family therapy for anorexia nervosa
MEED: Medical emergencies in eating disorders
NICE: National institute for health and care excellence
WFH: Weight for height
